# Development of Halochromic and Bioactive Polyvinyl Alcohol Films Enriched With *Kalanchoë blossfeldiana* Anthocyanins for Active and Intelligent Poultry Packaging

**DOI:** 10.1002/fsn3.71235

**Published:** 2025-11-28

**Authors:** Mahmoud Younis, Diaeldin Omer Abdelkarim, Mohamed Abdelbaset Salama, Mona Hussein Hassan, Reham M. Kamel, Mohamed Abdin, Yasmin Salama, Said El Harkaoui, Mohamed Reda Badr, Mahmoud Elsayed

**Affiliations:** ^1^ Chair of Dates Industry and Technology, Department of Agricultural Engineering, College of Food and Agricultural Sciences King Saud University Riyadh Saudi Arabia; ^2^ Department of Agricultural Engineering, Faculty of Engineering University of Khartoum Khartoum Sudan; ^3^ Agricultural Research Center Food Technology Research Institute Giza Egypt; ^4^ Agricultural Engineering Research Institute Agricultural Research Center Giza Egypt; ^5^ Faculty of Specific Education, Kafrelsheikh University Kafrelsheikh Egypt; ^6^ Max Rubner‐Institut (MRI) Department of Safety and Quality of Cereals, Working Group for Lipid Research Detmold Germany; ^7^ Food Science and Technology Department, Faculty of Agriculture Tanta University Tanta Egypt

**Keywords:** active packaging, antimicrobial and antioxidant properties, halochromic films, intelligent packaging, *Kalanchoë blossfeldiana* anthocyanins

## Abstract

This study developed halochromic polyvinyl alcohol (PVA) films incorporated with anthocyanins extracted from *Kalanchoë blossfeldiana* leaves (KBA1–KBA3) for intelligent food packaging. Anthocyanins were obtained using acidified ethanol and purified via column chromatography before incorporation into PVA via solvent casting. The addition of anthocyanins increased film thickness (0.124 to 0.170 mm), opacity, solubility, and swelling degree, while reducing water contact angle (86.21° to 68.31°) and enhancing hydrophilicity. Water vapor permeability increased, and color parameters (L*, a*, b*) shifted markedly with pigment loading. AFM revealed increased surface roughness, correlating with reduced tensile strength and flexibility. Films exhibited strong antioxidant activity (~70% DPPH and ABTS scavenging) and antimicrobial effects, with inhibition zones up to ~8 mm against *E. coli* and *S. aureus* . Thermal analysis indicated decreased stability with anthocyanin incorporation. Applied to chicken filet storage, the films showed clear, progressive halochromic color changes aligned with spoilage markers—pH increase (3.2 to 8.5), TVB‐N (> 30 mg N/100 g), and peroxide value (13.26 meq O_2_/kg) over 14 days. This is the first report using *K. blossfeldiana* anthocyanins in intelligent packaging, demonstrating their multifunctional potential for sustainable meat freshness monitoring.

## Graphical abstract

Anthocyanin‐enriched PVA films derived from *Kalanchoë blossfeldiana* demonstrated halochromic color changes in response to poultry spoilage, effectively correlating with pH, TVB‐N, and peroxide levels. These biodegradable films exhibited strong antioxidant (~70%) and antimicrobial activity (up to 8 mm inhibition zones). While higher pigment loading reduced thermal and mechanical stability, the films remained effective as intelligent, single‐use packaging for freshness monitoring.

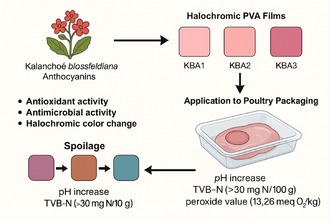

## 1 Introduction

The widespread reliance on petroleum‐based plastic packaging poses a critical global challenge due to the environmental concerns associated with its production and disposal. Traditional plastic packaging materials, typically sourced from detrimental petrochemicals, can be replaced with recyclable options made from environmentally beneficial ingredients like natural polymers and biodegradable additives (Samuel et al. 2024). As a result, there is growing interest in the development of sustainable packaging solutions that utilize natural and biological resources (Ibrahim et al. 2022). The current focus is on innovative food packaging, particularly those that are recyclable, multifunctional, and intelligent, as consumer awareness regarding food safety, sustainability, and environmental impact continues to rise (Yan et al. 2025). Comprehensive reviews of polysaccharide‐based intelligent packaging summarize recent advances in indicator chemistries, sensing architectures, and applications across meats and produce (Liu et al. 2025). These advanced packaging materials are capable of detecting fluctuations in pH to ensure continuous monitoring of food freshness, quality, and safety. Such systems often provide a visual indication, such as a color shift, allowing manufacturers, suppliers, and consumers to interpret product status. Among these technologies, halochromic biosensors are specifically designed for this purpose (Abdin, Naeem, and Aly‐Aldin 2024). Beyond polymer‐only indicators, hybrid systems have immobilized anthocyanins on metal–organic framework (MOF) carriers and coupled color readouts with back‐propagation neural networks (BPNN) to boost sensitivity and enable data‐driven freshness prediction (Liu et al. 2023).

Polyvinyl alcohol (PVA) is a water‐soluble, linear‐structured polymer that is recognized for being non‐toxic, non‐carcinogenic, and biocompatible. It exhibits excellent film‐forming properties, strong adhesion, and high emulsification capabilities, making it widely applicable across various industries. Additionally, PVA is the most extensively produced biodegradable synthetic polymer worldwide, owing to its remarkable mechanical properties, chemical resistance, and complete biodegradability (Korbag and Mohamed Saleh 2016; Younis et al. 2025). Incorporating plant extracts into biodegradable films plays a vital role in enhancing their functionality, enabling them to extend food shelf life or act as indicators of early spoilage (Abdin et al. 2023; Alshehri et al. 2024).

Anthocyanins are among the most commonly utilized natural extracts in smart packaging films. These phenolic compounds are responsible for the red, blue, and purple pigmentation in fruits and vegetables. This unique characteristic enables continuous monitoring of food quality. Furthermore, anthocyanins possess antimicrobial and antioxidant properties, which contribute to prolonging food shelf life (Ranganath 2022; Yousuf et al. 2016).

*Kalanchoë blossfeldiana* is a perennial succulent plant native to Madagascar and widely cultivated for its vibrant flowers and ornamental value. It belongs to the family Crassulaceae and is known for its drought tolerance, compact growth habit, and extended flowering period, making it popular in horticulture (Smith 2025). Beyond its ornamental appeal, *K. blossfeldiana* has attracted scientific interest due to its bioactive compounds, including flavonoids, triterpenoids, and phenolic acids, which contribute to its antioxidant and potential therapeutic properties (Danna 2024; Stefanowicz‐Hajduk et al. 2024). Several studies have reported anti‐inflammatory, antimicrobial, and cytotoxic activities from extracts of various Kalanchoë species, indicating their potential use in traditional and modern medicine (Mejía‐Méndez et al. 2023; Nascimento et al. 2023).

In recent years, global meat production has seen a notable increase, and this trend is expected to continue. However, animal‐derived food products are highly susceptible to microbial contamination and lipid oxidation due to their high fat and moisture content. These quality deteriorations can occur throughout different stages of the supply chain, including processing, storage, and distribution (Wood et al. 2024). Smart packaging technologies, incorporating indicators and sensors, are increasingly being used to provide real‐time monitoring of meat quality. These systems alert retailers and consumers to changes in freshness and microbial contamination (Nami et al. 2024). As customer demand for safe, high‐quality meat products increases, coupled with initiatives to reduce food waste, intelligent packaging technologies are poised to significantly impact the meat industry (Rebezov et al. 2024).

Therefore, this study aims to develop and characterize polyvinyl alcohol (PVA)‐based biodegradable films incorporated with anthocyanin extracts from *Kalanchoë blossfeldiana* leaves. The objective is to evaluate their physicochemical, optical, mechanical, antimicrobial, and antioxidant properties, as well as their potential halochromic behavior as freshness indicators in poultry packaging. By integrating natural pigments with known bioactive properties, the research seeks to create an intelligent and active packaging system that not only extends the shelf life of chicken filets but also provides a visual indication of spoilage, aligning with current trends in sustainable and smart food packaging technologies.

## 2 Materials and Methods

### 2.1 Materials

Polyvinyl alcohol (PVA), CAS No. 9002‐89‐5, with a purity of 94%, viscosity range of 22–30 cP, and a hydrolysis level of 99%–99.9% (mol), ethanol (analytical grade; Fisher Chemical), HCl (37%; Merck Millipore), formic acid (98%–100%; Merck Millipore), ABTS and DPPH (Sigma‐Aldrich, St. Louis, MO, USA) were used. Fresh chicken filets were purchased from a local market in Kafrelsheikh, Egypt. Freshly harvested bracts of the *Kalanchoë blossfeldiana* plant were collected from the Agricultural Research Center farm, Egypt.

### 2.2 Methods

#### 2.2.1 Extraction and Purification of *Kalanchoë anthocyanins* (KBA)

Fresh bracts were dried at 50°C for 20 h, milled, and sieved to pass a 60‐mesh screen. A 50.00 g portion of the powder was extracted with 500.0 mL 70% (v/v) ethanol acidified to pH 4.00 ± 0.05 with 1.0 M HCl at 25°C under magnetic stirring (400 rpm) for 15 h in amber glassware. The slurry was vacuum‐filtered and the filtrate centrifuged at 6000 rpm for 20 min at 4°C; the supernatant was concentrated by rotary evaporation at 45°C and 120 mbar to approximately one‐fifth of its initial volume. The concentrate was partitioned three times with an equal volume of ethyl acetate and the aqueous phase retained. The aqueous extract was loaded onto a pre‐equilibrated AXR column (5 × 52 cm; 0.5% v/v formic acid), washed with two bed volumes of the same solvent, and eluted with 60% ethanol at 2.0 mL·min^−1^. Fractions were monitored at 520 nm, pooled at A_520_ maxima, concentrated to remove solvent, and freeze‐dried to constant mass; yield was recorded as % (w/w) relative to the starting dry bract powder. Dried KBA was stored in amber vials at −20°C until use.

#### 2.2.2 Preparation of PVA/KBA Films

Polyvinyl alcohol (PVA; 99%–99.9% hydrolyzed; 22–30 cP) was dissolved at 3.50 g in 150.0 mL deionized water at 95°C under magnetic stirring (800 rpm) for 45 min, then cooled to 25°C for 2 h. Kalanchoë anthocyanin extract (KBA) was incorporated to obtain final loadings of 0.5%, 1.0%, and 1.5% (w/w relative to PVA) for formulations KBA1, KBA2, and KBA3, respectively, and the mixtures were stirred for 60 min. Glycerol (1.00 mL; final ≈0.28% w/v) was added as a plasticizer, and dispersions were degassed for 5 min. Casting was performed by dispensing 60.0 mL into 15‐cm glass Petri dishes, followed by drying at 50°C for 10 h. Dried films were peeled and conditioned at 25°C and 50% relative humidity for 48 h prior to cutting test specimens.

#### 2.2.3 Structural Characterization

##### 2.2.3.1 Morphological Characterization of Films

Surface topography of the films was analyzed by atomic force microscopy (AFM) using a Bruker Dimension Icon operated in tapping mode under ambient conditions. Film strips were cut with a clean steel blade and affixed to glass slides using double‐sided adhesive to ensure a flat, immobile surface; samples were equilibrated for 30 min prior to imaging. Scans were acquired at 5 × 5 μm (routine mapping) and 1 × 1 μm (high‐resolution detail) at 0.8–1.0 Hz line rate with the setpoint ≈70% of the free amplitude; the drive frequency was tuned at each location to the local resonance. Height and phase channels were recorded simultaneously. Images were processed in NanoScope Analysis with first‐order flattening (no filtering beyond plane/line removal). Roughness parameters—average roughness (Ra) and root‐mean‐square roughness (Rq)—were computed from five randomly selected areas per film, *n* = 3 films per formulation, and reported as mean ± SD. Locations showing edge artifacts, particulate debris, or obvious feedback loss were excluded a priori. Scale bars are included on all panels.

#### 2.2.4 Physicochemical Properties

##### 2.2.4.1 Characterization of Films Thickness, Solubility, Swelling Degree

Film thickness was measured at six random points using a micrometer. Solubility was assessed by drying five 2 × 2 cm^2^ film pieces to constant weight (M1), immersing in water for 24 h, filtering insoluble residues, and drying again (M2 and M3). The swelling index was determined using air‐dried filter papers weighed before (M3) and after water exposure (M4).

(1) $$\text{Solubility}\;\left(\%\right)=\left(\frac{M2-M3}{M2}\right)\times 100$$

(2) $$\text{Swelling degree}\;\left(\%\right)=\left(\frac{M4-M3}{M3}\right)\times 100$$

##### 2.2.4.2 Determination of Films Water Vapor Permeability ( *WVP* )

A conical flask containing 15 mL distilled water was sealed with the film. Flasks were placed in a desiccator at 25°C and 50% RH. Weight changes were recorded every 2 h for 12 h.

(3) $$\mathrm{WVP}=\frac{\mathrm{\Delta w}.f}{A.t.\mathrm{\Delta P}}$$

where Δ*w* is the weight gain, f is film thickness, A is the surface area, t is time, and ΔP is the vapor pressure difference.

##### 2.2.4.3 Characterization of Films Color and Opacity

Color attributes (L, a, b*) were measured using a Konica Minolta colorimeter. Four readings per sample were averaged. Color difference relative to the control (pure PVA) was calculated as

(4) $${\mathrm{\Delta E}}^{*}={\left[{\left({\mathrm{\Delta L}}^{*}\right)}^{2}+{\left({\mathrm{\Delta a}}^{*}\right)}^{2}+{\left({\mathrm{\Delta b}}^{*}\right)}^{2}\right]}^{1/2}$$

Opacity was determined using a UV–Vis spectrophotometer (600 nm):

(5) $$\text{Opacity}=\frac{\mathrm{Ab}600}{\text{Thickness}\;\left(\mathrm{mm}\right)}$$

##### 2.2.4.4 Water Contact Angle Measurement of Films

The surface wettability of PVA/EPA films was assessed following the method described by Kraisit et al. (2015) using a HARKE‐SPCAX1 optical contact angle goniometer. The sessile drop method was employed, wherein each film was affixed to a glass slide with adhesive tape, and a small droplet of distilled water was gently placed on the film surface. The contact angle was determined by capturing and analyzing the droplet profile. Each sample was measured in quadruplicate to ensure accuracy and reproducibility.

##### 2.2.4.5 Mechanical Properties of Films

Tensile strength (TS) and elongation at break (EB) of the halochromic films were determined using an Instron 5567 universal testing machine. Samples measuring 2 × 12 cm were mounted between the grips, with a crosshead speed of 100 mm/min and a gauge length of 30 mm. TS and EB were calculated using the following equations:

(6) $$\mathrm{TS}\;\left(\mathrm{MPa}\right)=\frac{\mathrm{Fm}}{\mathrm{Th}\times \mathrm{Wi}}$$

(7) $$\mathrm{EB}\;\left(\%\right)=\left(\frac{\mathrm{Il}-L0}{L0}\right)\times 100$$

where *Fm* = maximum force (*N*), *Th* = thickness (mm), *Wi* = width (mm), *Il* = length at break (mm), *L0* = initial length (mm).

#### 2.2.5 Functional Activity

##### 2.2.5.1 Antioxidant Activity Evaluation of Films

The antioxidant activity of the films was examined using DPPH and ABTS radical scavenging assays with slight adaptations from the method reported in (Abdin et al. 2024).

For DPPH, 30 mg of film was immersed in 5 mL of distilled water, then extracted with another 5 mL. A 1 mL aliquot of the extract was mixed with 1 mL of 0.1 mM DPPH methanolic solution and kept in the dark for 30 min. The absorbance at 517 nm was measured, and the scavenging activity was computed as:

(8) $$\begin{array}{c} \text{DPPH radical scavenging activity}\;\left(\%\right)\\ =\left(\frac{\mathrm{Abs}\;\text{control}-\mathrm{Abs}\;\text{films}}{\mathrm{Abs}\;\text{control}}\right)\times 100 \end{array}$$

For ABTS, 30 mg of film was soaked in 8 mL of water and extracted with another 8 mL. ABTS radicals were generated by reacting 7 mM ABTS with 145 mM potassium persulfate, incubated in the dark for 12 h. The working solution was diluted with PBS (pH 7.4) to an absorbance of ~0.8. The reaction mixture (20 μL of extract +1980 μL ABTS solution) was measured at 734 nm (Abdin et al. 2023). The ABTS radical scavenging percentage was calculated as:

(9) $$\text{ABTS radical scavenging activity}\;\left(\%\right)=\left(1-\frac{{\mathrm{Abs}}_{1}-{\mathrm{Abs}}_{2}}{{\mathrm{Abs}}_{0}}\right)$$

where Abs_0_ = absorbance of ABTS solution, Abs_1_ = absorbance with film extract, Abs_2_ = absorbance with PBS.

##### 2.2.5.2 Antimicrobial Activity Evaluation of Films

Antibacterial activity of the films was evaluated by disc diffusion on Mueller–Hinton agar (MHA) against *Escherichia coli* ATCC 25922 and *Staphylococcus aureus* ATCC 6538, adapted from CLSI principles. Overnight cultures grown in Mueller–Hinton broth (MHB, 37°C, 200 rpm) were adjusted to 0.5 McFarland (≈1 × 10^8^ CFU·mL^−1^, verified by plate count). Each MHA plate was inoculated by evenly spreading 100 μL of the standardized suspension (≈1 × 10^7^ CFU·plate^−1^) and allowed to absorb for 10 min at room temperature.

Film specimens were punched into 10 mm‐diameter discs and conditioned at 25°C, 50% RH for 24 h. Discs of PVA–KBA1, PVA–KBA2, PVA–KBA3, and blank PVA (negative control) were gently placed onto the inoculated agar surface (one disc per quadrant), ensuring full contact and ≥ 24 mm spacing between disc centers to avoid overlapping zones. Plates were incubated inverted at 37°C for 24 h.

#### 2.2.6 Application in Chicken Filet Packaging

Fresh chicken filets (200 g) were placed in transparent polypropylene containers (15 × 15 cm). Halochromic films (7 cm diameter) were adhered to the inner lid using adhesive. The packages were partially sealed and stored at 5°C for 14 days. Color transitions in the films were visually monitored and documented using a smartphone camera.

##### 2.2.6.1 Assessment of pH, TVB‐N, and Peroxide Value (PV)

For pH determination, 10 g of filet sample was homogenized with 90 mL distilled water and filtered through Whatman No. 2 paper. The filtrate was measured using an Orion Star A211 digital pH meter (Thermo Scientific, Indonesia). TVB‐N was determined following the protocol described by Jouki et al. (2014). To evaluate PV, 2 g of sample was mixed with a solution of acetic acid and chloroform (12:18 v/v). Then, 1 mL of potassium iodide was added, shaken, and incubated in the dark for 5 min. Subsequently, 30 mL of distilled water and 1 mL starch indicator were added, and the mixture was titrated with 0.01 N sodium thiosulfate. PV was calculated using:

(10) $$\mathrm{PV}=\frac{\left(V_{1}-V_{2}\right)\times N\times 1000}{W}$$

where *V*_1_ = titration volume for sample (mL), *V*_2_ = titration volume for blank (mL), *N* = normality of Na_2_S_2_O_3_, W = sample weight (g).

#### 2.2.7 Statistical Analysis

All results were statistically analyzed using SPSS software (version 16.0, SPSS Inc., Chicago, IL, USA). Tukey's Honest Significant Difference (HSD) test was employed to determine significant differences at a 95% confidence level.

## 3 Results and Discussion

### 3.1 Morphological Characterization of PVA and Anthocyanin‐Enhanced Films

**FIGURE 1:**
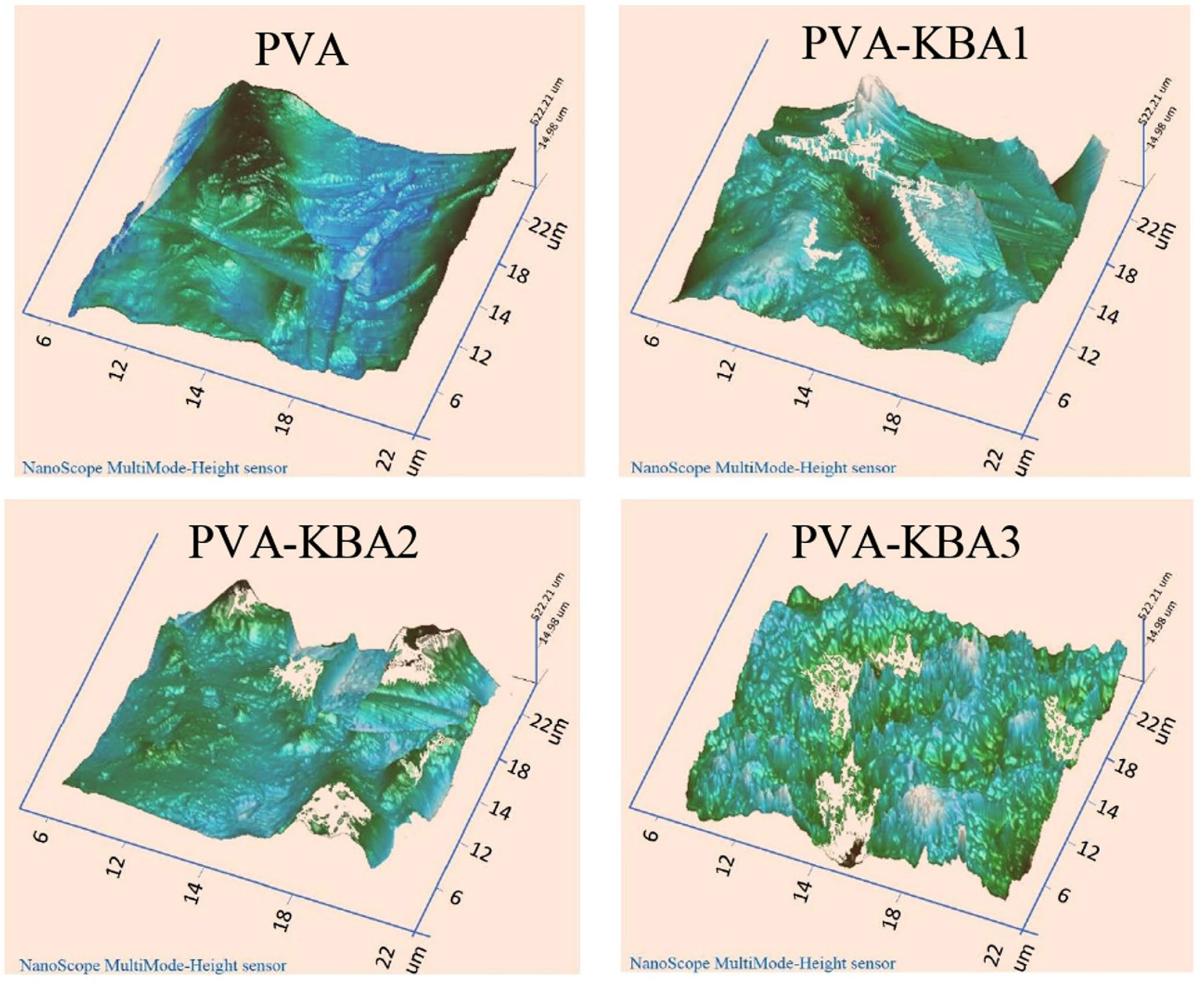
Atomic force microscopy (AFM) of control PVA films and PVA films enriched with *Kalanchoë blossfeldiana* anthocyanins (KBA) at different concentrations.

The AFM images demonstrate the effect of incorporating increasing concentrations of anthocyanin pigment extracted from *Kalanchoë blossfeldiana* leaves into PVA‐based biodegradable films (Figure 1). The pure PVA film exhibits a smooth and uniform surface, indicative of a homogeneous polymer matrix. However, as anthocyanin is added, the surface roughness progressively increases, with the highest concentration (KBA3) showing significant irregularities, likely due to pigment aggregation. At lower concentrations (KBA1 and KBA2), the anthocyanins appear to form interactions with the PVA matrix through hydrogen bonding, resulting in better dispersion and moderate changes to the film's morphology. Structure–property implications (mechanical trade‐offs) are treated in Section 3.6. Thus, while low concentrations may balance improved bioactivity with structural stability, higher concentrations risk compromising film performance. These findings are consistent with studies by Yücetürk et al. (2024) and Ezati et al. (2023), which highlight the trade‐offs between functional enhancements and structural integrity in biopolymer films with high pigment loads.

### 3.2 Physical, Opacity, and Color Properties of PVA and Anthocyanin‐Enhanced Films

The thickness of the films increases progressively with higher anthocyanin concentrations, from 0.124 mm (pure PVA) to 0.170 mm (PVA‐KBA3) (Table 1). This increase is likely due to the incorporation of anthocyanins, which introduce additional molecular interactions and structural modifications in the PVA matrix. The phenolic compounds in anthocyanins may create a more compact or reinforced polymer network, leading to an increase in film thickness (Yong and Liu 2020). Additionally, anthocyanin molecules can interfere with PVA chain alignment, contributing to variations in film morphology, as also observed in the AFM images where surface roughness increased with anthocyanin content.

**TABLE 1:** Physicochemical properties of control PVA films and PVA films enriched with *Kalanchoë blossfeldiana* anthocyanins (KBA) at different oncentrations.

Parameters	Control films PVA	Films enhanced with anthocyanins		
		PVA/KBA1	PVA/KBA2	PVA/KBA3
Thickness (mm)	0.124 ± 0.003^c^	0.125 ± 0.002^c^	0.148 ± 0.001^b^	0.170 ± 0.002^a^
Solubility (%)	6.25 ± 0.58^d^	17.68 ± 0.74^c^	21.48 ± 0.55^b^	27.91 ± 0.65^a^
Swelling degree (%)	17.51 ± 0.52^d^	20.19 ± 0.58^c^	25.65 ± 0.98^b^	35.51 ± 0.87^a^
*WVP* (× 10^−10^ gm^−1^ s‐1 pa^−1^)	0.264 ± 0.000^c^	0.597 ± 0.002^b^	0.611 ± 0.002^b^	1.005 ± 0.004^a^
Opacity	0.854 ± 0.34^d^	1.215 ± 0.11^c^	1.864 ± 0.31^b^	2.325 ± 0.39^a^
Color parameters				
L*	92.15 ± 0.54^a^	70.64 ± 0.71^b^	39.25 ± 0.47^c^	20.54 ± 0.58^d^
a*	0.64 ± 0.03^d^	30.65 ± 0.47^c^	37.55 ± 0.86^b^	50.74 ± 0.46^a^
b*	0.20 ± 0.02^d^	24.47 ± 0.08^c^	39.06 ± 0.33^b^	48.54 ± 0.74^a^
ΔE	—	44.18 ± 0.54^c^	75.31 ± 0.68^b^	99.87 ± 0.71^a^

*Note:* Values are means ± SD. Means having the different case letter (s) within a row are significantly different at *p* ≤ 0.05. Abbreviation: WVP, water vapor permeability.

The solubility and swelling degree of the films show a significant increase with anthocyanin incorporation. Pure PVA films exhibit a low solubility (6.25%), while PVA‐KBA3 films show the highest solubility (27.91%). Similarly, swelling increases from 17.51% (PVA) to 35.51% (PVA‐KBA3) (Table 1). The increase in solubility and swelling degree is due to the presence of hydrophilic phenolic compounds in anthocyanins, which enhance water interactions, leading to greater water uptake and swelling capacity (Grgić et al. 2020). This trend aligns with the water contact angle (WCA) results, where increasing anthocyanin concentration led to lower contact angles, confirming higher hydrophilicity.

Water vapor permeability (WVP) increases significantly with anthocyanin incorporation, from 0.264 (pure PVA) to 1.005 × 10^−10^ g.m^−1^ s^−1^ Pa^−1^ (PVA‐KBA3) (Table 1). The increase in WVP can be attributed to the disruption of the PVA matrix by anthocyanins, which may reduce polymer cohesion and increase free volume within the film, allowing for greater moisture transmission (Zhang et al. 2024). This finding is consistent with the mechanical properties results, where higher anthocyanin concentrations led to reduced tensile strength, indicating weaker intermolecular forces within the film structure.

Opacity values increase significantly with anthocyanin incorporation, from 0.854 (pure PVA) to 2.325 (PVA‐KBA3) (Table 1). This increase is expected as anthocyanins are natural pigments that absorb light in the visible spectrum, reducing film transparency. A higher opacity is beneficial for active packaging applications, as it helps protect food products from light‐induced oxidative degradation (Passaretti et al. 2019). Opacity rise (0.854 → 2.325 mm^−1^) and strong shifts in L, a, b* are consistent with anthocyanin‐film studies using red cabbage or purple sweet potato extracts, which likewise report pronounced darkening and red shift at ≥ 1% pigment (He and Giusti 2010; Singh et al. 2020; Chiu and Yang 2024).

The colorimetric properties of the films were significantly influenced by the incorporation of *Kalanchoë blossfeldiana* anthocyanins (Table 1). The lightness value (L*) markedly decreased from 92.15 in pure PVA films to 20.54 in PVA‐KBA3 films, indicating a pronounced darkening effect with increasing pigment concentration. This trend is attributed to the intense coloration of anthocyanins, which absorb light in the visible region and consequently reduce light transmission through the film matrix (Abedi‐Firoozjah et al. 2022). Concurrently, the a* values (red‐green axis) increased significantly from 0.64 in the control to 50.74 in PVA‐KBA3, confirming the strong red pigmentation imparted by anthocyanins. Similarly, the b* values (yellow‐blue axis) rose from 0.20 to 48.54, suggesting a shift toward orange‐red hues. ΔE increased monotonically with pigment loading: KBA1 = 44.18, KBA2 = 75.31, KBA3 = 99.87. Given common perceptibility thresholds (ΔE*≈2), all formulations exhibit readily discernible color with very high contrast, supporting sensitive visual discrimination across loadings.

The observed results are consistent with other studies where anthocyanin‐rich extracts, such as those from red cabbage, purple sweet potato, or black rice, were incorporated into biopolymer films and caused significant alterations in color attributes (Chiu and Yang 2024; Singh et al. 2020). These findings underscore the potential of KBA pigments not only for visual appeal but also for functional smart packaging applications.

### 3.3 Water Contact Angle of PVA and Anthocyanin‐Enhanced Films

**FIGURE 2:**
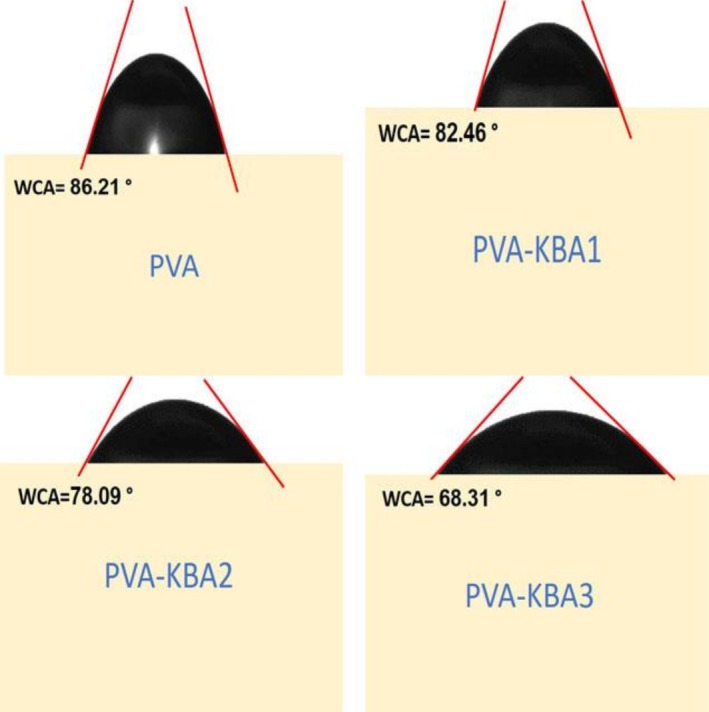
Water contact angle of control PVA films and PVA films enriched with *Kalanchoë blossfeldiana* anthocyanins (KBA) at different concentrations.

The water contact angle (WCA) measurements of the biodegradable films reveal a decreasing trend with increasing anthocyanin content, indicating an enhancement in the hydrophilic nature of the films (Figure 2). The pure PVA film exhibits a WCA of 86.21°, reflecting its moderate hydrophilicity due to hydroxyl (‐OH) groups in the polymer structure. With the incorporation of anthocyanins, the WCA progressively decreases: PVA‐KBA1 has a WCA of 82.46°, PVA‐KBA2 has a WCA of 78.09°, and PVA‐KBA3 records the lowest WCA at 68.31°. This trend suggests that anthocyanins enhance the wettability of the films by increasing the presence of polar functional groups on the surface.

The reduction in WCA with increasing anthocyanin content is likely due to the hydrophilic nature of anthocyanins, which contain multiple hydroxyl (‐OH) groups that interact with water molecules (Wu and Li 2022). At lower concentrations (PVA‐KBA1), these interactions are limited, leading to a slight decrease in WCA. However, as the anthocyanin content increases (PVA‐KBA2 and PVA‐KBA3), the film surface becomes more enriched with hydrophilic functional groups, enhancing water affinity and reducing the contact angle. This trend aligns with previous studies where the incorporation of phenolic compounds into polymer matrices resulted in improved hydrophilicity (Abdin et al. 2021; Akhtar et al. 2018; Riaz et al. 2020). Additionally, the increase in surface roughness observed in the AFM images of higher anthocyanin‐loaded films could further contribute to the decrease in WCA by enhancing water absorption through capillary effects (Abdin, Naeem, Elmahdy, and Ali 2024). These findings indicate that anthocyanin‐modified PVA films could have potential applications in food packaging, where controlled hydrophilicity can influence moisture interactions and biodegradability.

The WCA decline is similar to the drops that are frequently observed when the surface becomes rougher and enriched with phenolic hydroxyls (Akhtar et al. 2018; Riaz et al. 2020; Wu and Li 2022).

Controlled relative humidity (RH) stability was not quantified here. A planned follow‐up will condition films at 33%, 57%, 75%, and 97% RH (25°C; saturated salt chambers) and reassess TS/EB, WVP, WCA, and color/ΔE* to map moisture–plasticization effects.

### 3.4 TGA Properties of PVA and Anthocyanin‐Enhanced Films

**FIGURE 3:**
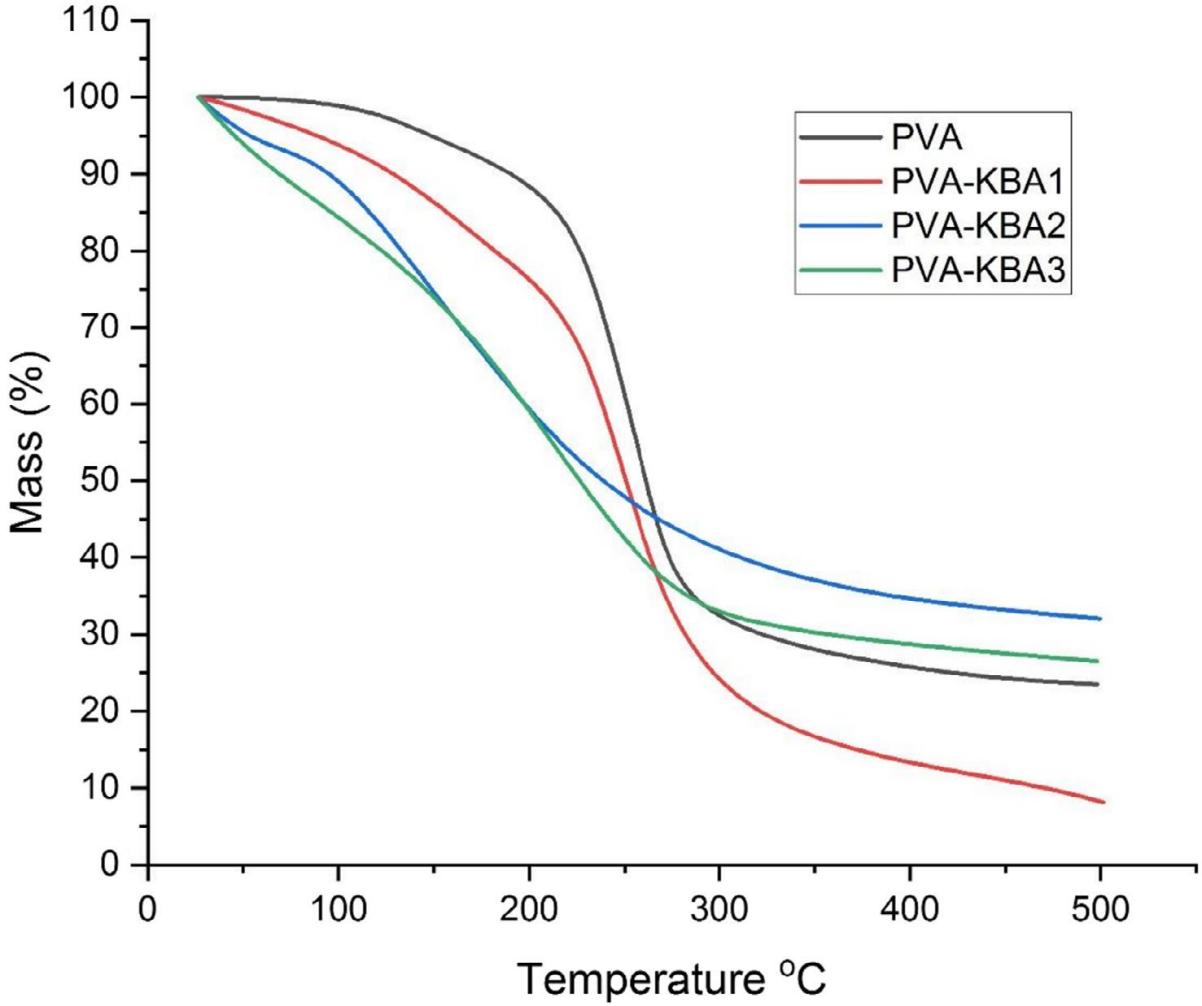
Thermogravimetric analysis (TGA) of control PVA films and PVA films enriched with *Kalanchoë blossfeldiana* anthocyanins (KBA) at different concentrations.

The thermogravimetric analysis (TGA) results reveal the thermal stability of the biodegradable films and how anthocyanin incorporation affects their degradation behavior (Figure 3). The pure PVA film exhibits the highest thermal stability, with significant weight loss occurring in two main stages: an initial mass loss below 150°C due to moisture evaporation and a major degradation event around 250°C–450°C, attributed to the breakdown of the polymer backbone. The incorporation of anthocyanins alters this thermal degradation pattern, with increasing anthocyanin concentrations leading to a more pronounced mass loss at lower temperatures. Among the modified films, PVA‐KBA1 retains relatively higher thermal stability, while PVA‐KBA2 and PVA‐KBA3 degrade more rapidly, showing significant weight loss at lower temperatures.

The decrease in thermal stability with higher anthocyanin concentrations can be attributed to the thermal decomposition of anthocyanins and their influence on the PVA matrix. Anthocyanins contain phenolic and hydroxyl groups that can disrupt the polymer network, lowering the energy required for degradation (Cai et al. 2022; Kossyvaki et al. 2022). Additionally, the increased hydrophilicity of anthocyanin‐enriched films, as observed in water contact angle (WCA) measurements, suggests a higher moisture retention capacity, which can accelerate thermal degradation at lower temperatures (Bian et al. 2023). The presence of anthocyanins may also introduce phase separation within the polymer matrix, weakening the polymer‐filer interaction and promoting early degradation. These findings indicate that while anthocyanins enhance the functional properties of PVA films, their impact on thermal stability should be considered in applications where high‐temperature resistance is required. Consistent DTG peak shifts (Section 3.5) corroborate the reduced stability; detailed temperatures are reported there.

Phenolic‐modified matrices also frequently exhibit smaller DTG peaks and an earlier commencement of mass loss upon the addition of pigment (Mohammadalinejhad and Kurek 2021; Kossyvaki et al. 2022).

### 3.5 The Derivative Thermogravimetric Analysis (DTG) of PVA and Anthocyanin‐Enhanced Films

**FIGURE 4:**
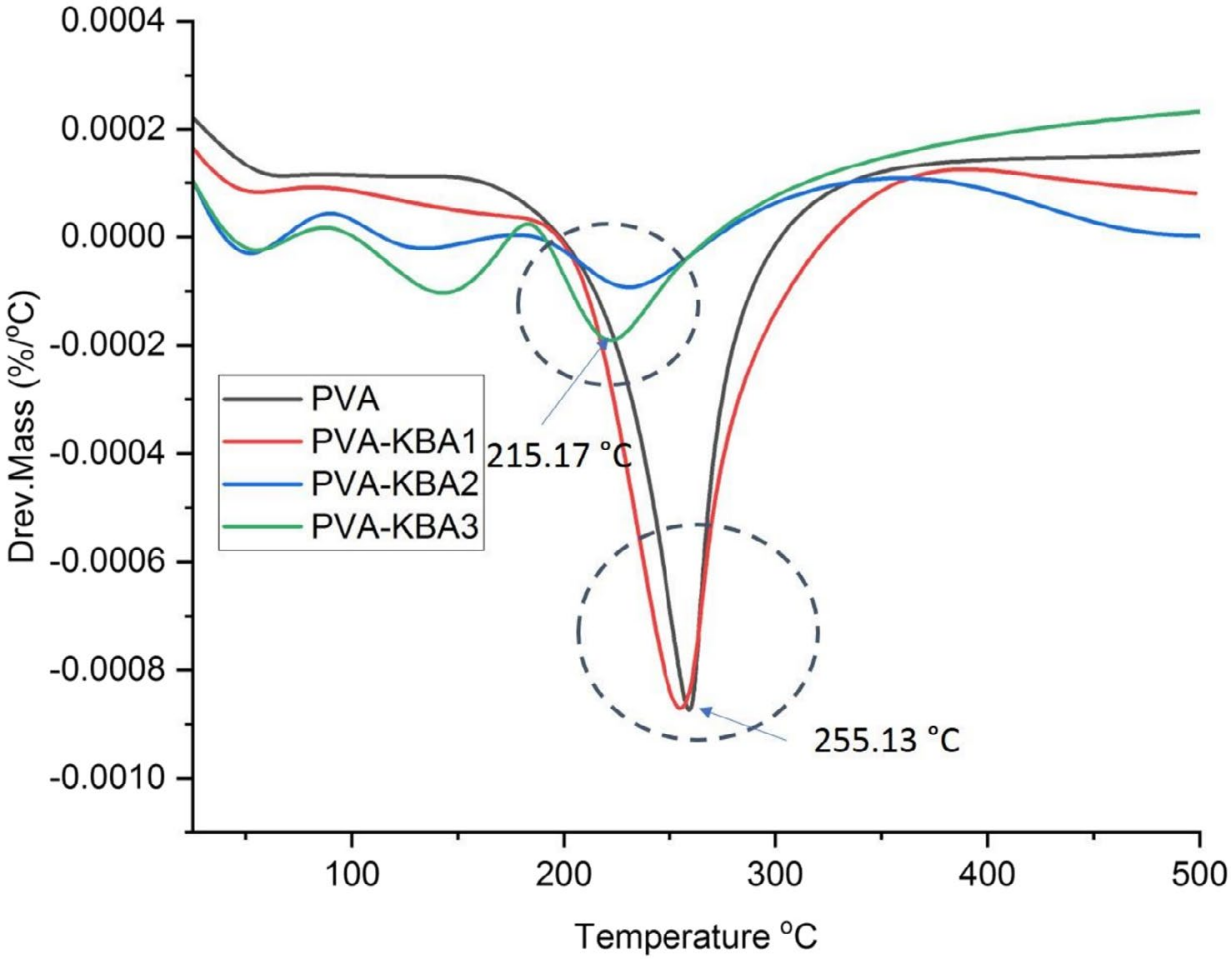
Derivative thermogravimetric (DTG) of control PVA films and PVA films enriched with *Kalanchoë blossfeldiana* anthocyanins (KBA) at different concentrations.

The derivative thermogravimetric analysis (DTG) curves provide detailed insights into the thermal decomposition behavior of the PVA‐based biodegradable films with increasing anthocyanin concentrations (Figure 4). The pure PVA film exhibits two major degradation peaks: the first at ~215.17°C, likely associated with the breakdown of low‐molecular‐weight components, and the second at ~255.13°C, corresponding to the main degradation of the polymer backbone. These peaks represent the primary thermal degradation stages of PVA, involving dehydration, side‐chain elimination, and polymer chain scission.

With anthocyanin incorporation, significant shifts in thermal degradation behavior are observed. The PVA‐KBA1 film retains a degradation pattern close to pure PVA but shows a slight reduction in peak temperatures, indicating minor structural disruption. However, PVA‐KBA2 and PVA‐KBA3 exhibit even lower degradation peaks, suggesting reduced thermal stability.

The trend observed in DTG aligns with the TGA results, confirming that higher anthocyanin concentrations increase polymer instability. Additionally, the increased surface roughness seen in AFM images suggests phase separation, which weakens polymer–polymer interactions, thereby reducing the films' overall thermal resistance. Studies by Yücetürk et al. (2024) also indicate that phenolic‐enriched biopolymer films tend to degrade earlier due to secondary interactions between plant‐based compounds and polymer backbones. Thus, while anthocyanin‐enriched PVA films enhance functional properties such as antioxidant activity and antimicrobial efficacy, their thermal stability decreases, necessitating careful formulation adjustments depending on the target application.

### 3.6 Mechanical Properties of PVA and Anthocyanin‐Enhanced Films

**FIGURE 5:**
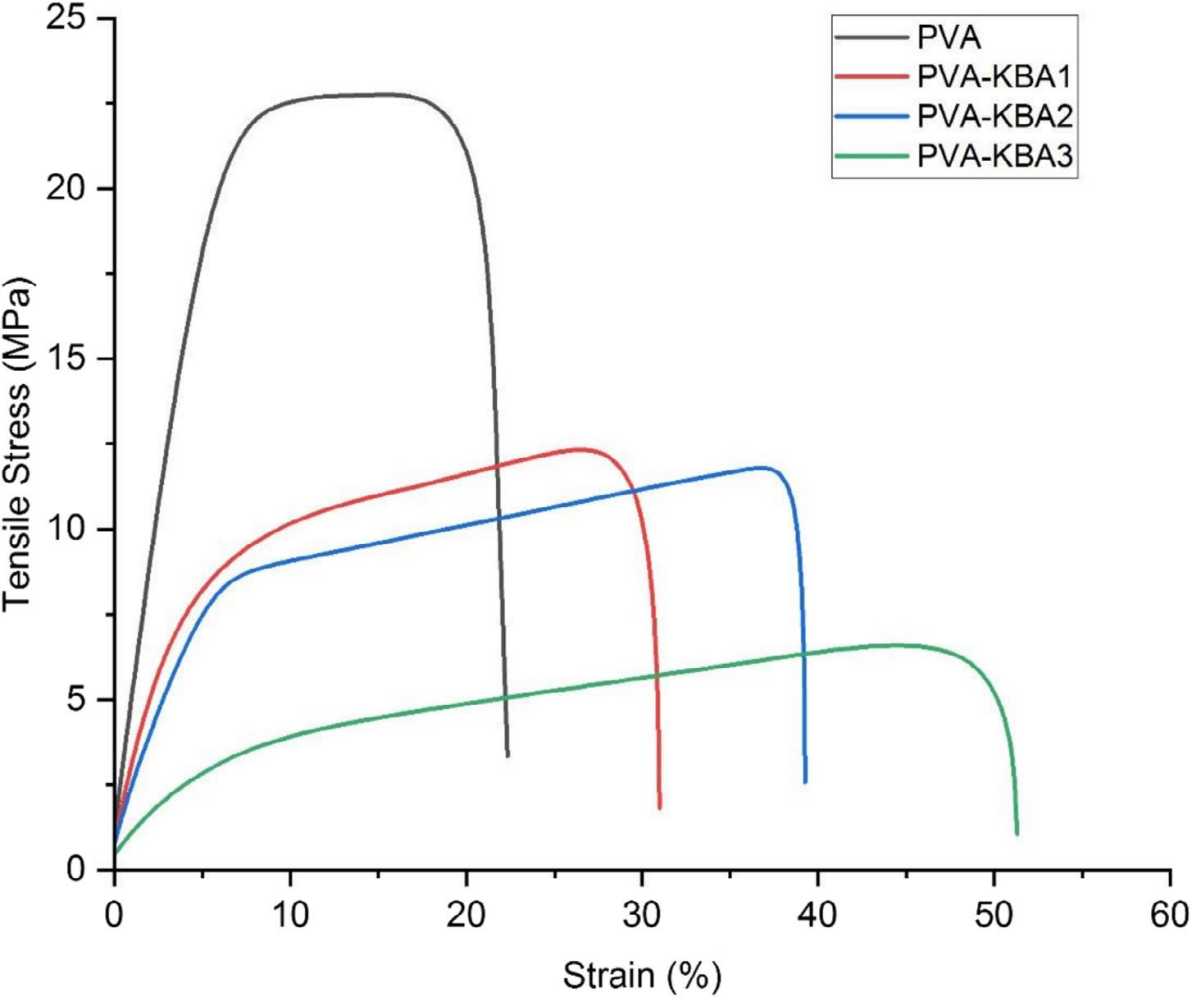
Mechanical properties of control PVA films and PVA films enriched with *Kalanchoë blossfeldiana* anthocyanins (KBA) at different concentrations.

The mechanical properties of the biodegradable films, as shown in the tensile stress–strain curves, indicate the influence of anthocyanin pigment concentration on the strength and flexibility of the films (Figure 5). The pure PVA film demonstrates the highest tensile stress (~22 MPa) and moderate elongation at break (~30%), which is characteristic of a homogeneous polymer matrix with well‐organized molecular interactions. However, as anthocyanin pigment is added, both tensile strength and elongation at break decrease, with the most significant reduction observed in PVA‐KBA3, which has a tensile stress of ~6 MPa and elongation at break of ~20%. At higher loadings, pigment aggregation and local phase separation introduce stress concentrators and weaken filer–matrix coupling, consistent with the roughness increase observed by AFM results.

The observed decline in mechanical performance with increasing anthocyanin concentration is likely due to disruptions in the PVA polymer network (Qi and Li 2024; Xu et al. 2022). At low concentrations (PVA‐KBA1), anthocyanins may interact favorably with PVA through hydrogen bonding, partially maintaining the structural integrity of the matrix, as reported by Xu et al. (2022). However, higher anthocyanin concentrations (PVA‐KBA2 and PVA‐KBA3) can lead to phase separation, aggregation of pigment molecules, and weak filer‐matrix interactions, which introduce stress concentration points and reduce the film's mechanical strength (Li et al. 2022). Furthermore, the increased surface roughness observed in the AFM images correlates with these mechanical trends, as rougher surfaces often indicate heterogeneity and reduced matrix cohesion.

Despite the decrease in mechanical properties, films with higher anthocyanin content may still be suitable for applications where moderate mechanical strength is acceptable, such as food packaging, given their superior antioxidant and antimicrobial functionalities. Optimization of anthocyanin loading is essential to balance mechanical integrity with functional properties. Similar findings have been reported in studies exploring phenolic compound‐enriched biopolymer films, where the trade‐off between bioactivity and mechanical strength was highlighted (Abdin, Naeem, Elmahdy, and Ali 2024; Alshehri et al. 2024; Gomaa et al. 2022).

The observed decrease in tensile strength (≈22 → ≈6 MPa from PVA to PVA‐KBA3; EB≈30% →≈20%) was consistent with studies for PVA or polysaccharide films loaded with anthocyanins, where phenolic clustering degrades the network at higher loadings. For instance, at ≥ 1% w/w pigment, Xu et al. (2022) and Li et al. (2022) found 20%–70% TS decreases while keeping EB between 15% and 40%. As a result, our values are within the usual range for smart‐indicator films that compromise functionality for strength.

### 3.7 Antioxidant Activity of PVA and Anthocyanin‐Enhanced Films

**FIGURE 6:**
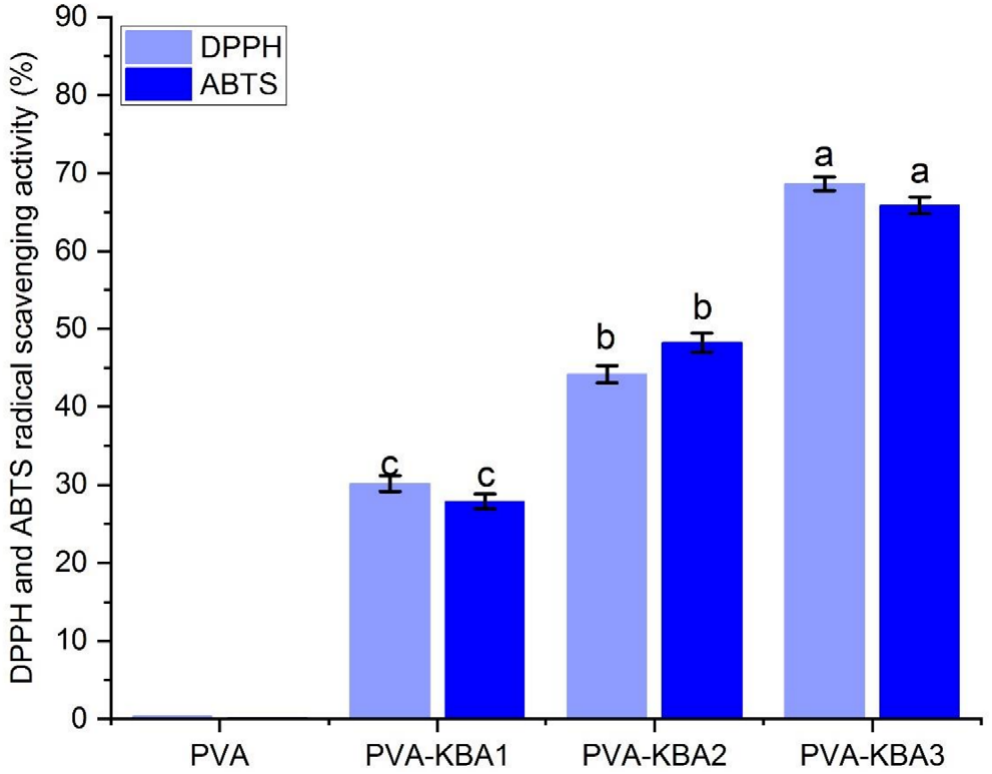
Antioxidant activity of control PVA films and PVA films enriched with *Kalanchoë lossfeldiana* anthocyanins (KBA) at different concentrations.

The antioxidant activity of the films, as assessed by DPPH and ABTS radical scavenging assays, reveals a concentration‐dependent increase with anthocyanin incorporation (Figure 6). The pure PVA film shows negligible antioxidant activity (< 5% for both DPPH and ABTS assays), confirming the lack of intrinsic radical‐scavenging properties in PVA. The addition of anthocyanin pigment extracted from *Kalanchoë blossfeldiana* leaves significantly enhances the antioxidant properties. PVA‐KBA1 shows approximately 30% scavenging activity for both DPPH and ABTS assays, while PVA‐KBA2 exhibits 50% scavenging activity. The highest antioxidant activity is observed for PVA‐KBA3, with values reaching approximately 70% for both assays.

The antioxidant activity results, combined with the antimicrobial and morphological data from the previous analyzes, provide a comprehensive understanding of the functional properties of the PVA‐based films enriched with anthocyanins from *Kalanchoë blossfeldiana* leaves. The pure PVA film consistently shows negligible activity in all tested parameters (antioxidant and antimicrobial), as it lacks bioactive compounds. However, the incorporation of anthocyanins significantly enhances both the antioxidant and antimicrobial properties, with the magnitude of improvement directly linked to the anthocyanin concentration.

The 50%–80% radical‐scavenging and 6–10 mm zones reported for anthocyanin‐rich indicator films at similar loadings are consistent with the ~70% DPPH/ABTS scavenging and inhibition zones up to ~8 mm (Mattioli et al. 2020; Sanhueza et al. 2017; Abdin et al. 2023). These trends are consistent with phenolic radical‐quenching behavior reported for anthocyanin‐enriched films.

### 3.8 Antimicrobial Activity of PVA and Anthocyanin‐Enhanced Films

**FIGURE 7:**
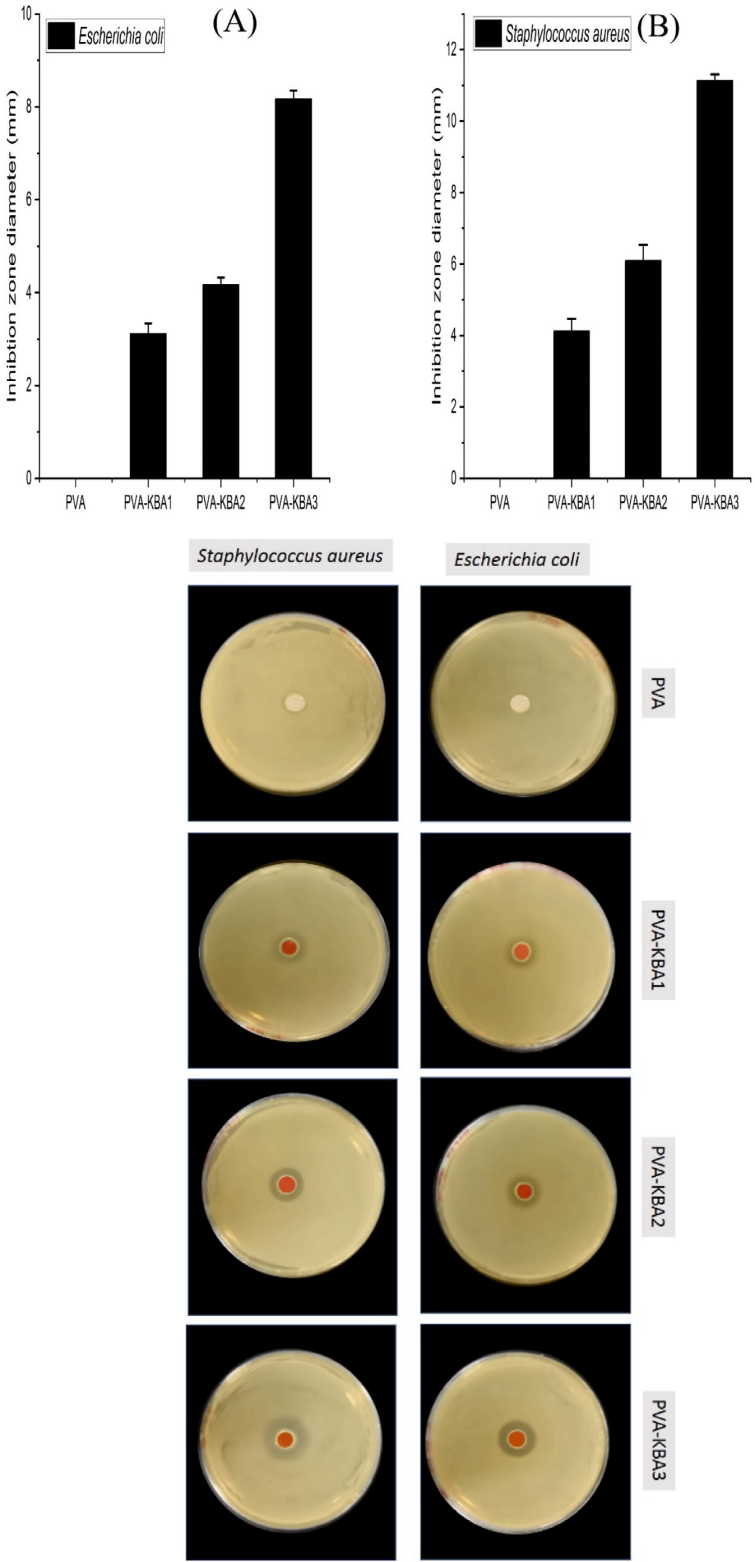
Antimicrobial activity of control PVA films and PVA films enriched with *Kalanchoë blossfeldiana* anthocyanins (KBA) at different concentrations against *Escherichia coli* (A) and *Staphylococcus aureus* (B).

The inhibition zone data clearly demonstrate the antimicrobial effect of biodegradable films against *Escherichia coli* with varying anthocyanin pigment concentrations extracted from *Kalanchoë blossfeldiana* leaves (Figure 7a). The pure PVA film shows no inhibition zone, indicating that PVA alone lacks intrinsic antimicrobial activity. In contrast, the addition of anthocyanins enhances the antimicrobial performance, with inhibition zone diameters increasing progressively as the concentration of anthocyanins rises. The PVA‐KBA1 film exhibits a modest inhibition zone, PVA‐KBA2 shows a greater zone, and PVA‐KBA3 achieves the highest inhibition zone, reaching approximately 8 mm.

Higher anthocyanin concentrations provide a larger pool of phenolic compounds capable of inhibiting microbial growth. These findings align with studies that have demonstrated the dose‐dependent antimicrobial effects of phenolic compounds in biopolymer matrices (Abdin et al. 2023; Abdin, Naeem, Elmahdy, and Ali 2024). Thus, incorporating anthocyanins into PVA films not only enhances their functional properties but also provides a sustainable and effective method to combat microbial contamination.

The inhibition zone results against *Staphylococcus aureus* demonstrate a clear trend in antimicrobial efficacy as anthocyanin pigment concentrations increase in PVA‐based biodegradable films (Figure 7b). Pure PVA films show no inhibition, confirming that PVA alone lacks antimicrobial properties. The addition of anthocyanins enhances antimicrobial activity, with the inhibition zone increasing progressively from PVA‐KBA1 to PVA‐KBA3. The largest inhibition zone is observed for PVA‐KBA3, indicating that higher anthocyanin concentrations substantially improve the antimicrobial effect.

*S. aureus* , being a Gram‐positive bacterium, is more susceptible to phenolic compounds due to its less complex cell wall compared to Gram‐negative bacteria like *E. coli* . The increasing inhibition zone correlates with the higher anthocyanin concentration, which likely enhances the release of bioactive compounds from the films. Similar findings have been reported in studies where phenolic‐enriched biopolymer films exhibited dose‐dependent antimicrobial effects (Morshdy et al. 2023; Sanhueza et al. 2017; Suparno et al. 2024). These results highlight the potential of anthocyanin‐enriched PVA films as effective antimicrobial packaging materials for controlling foodborne pathogens.

The dose‐dependent inhibition likely stems from phenolic‐mediated membrane disruption and oxidative stress; *S. aureus* typically shows greater susceptibility than Gram‐negative *E. coli* due to cell‐wall architecture.

### 3.9 Changes in Halochromic Colors During Storage of Chicken Filet

**FIGURE 8:**
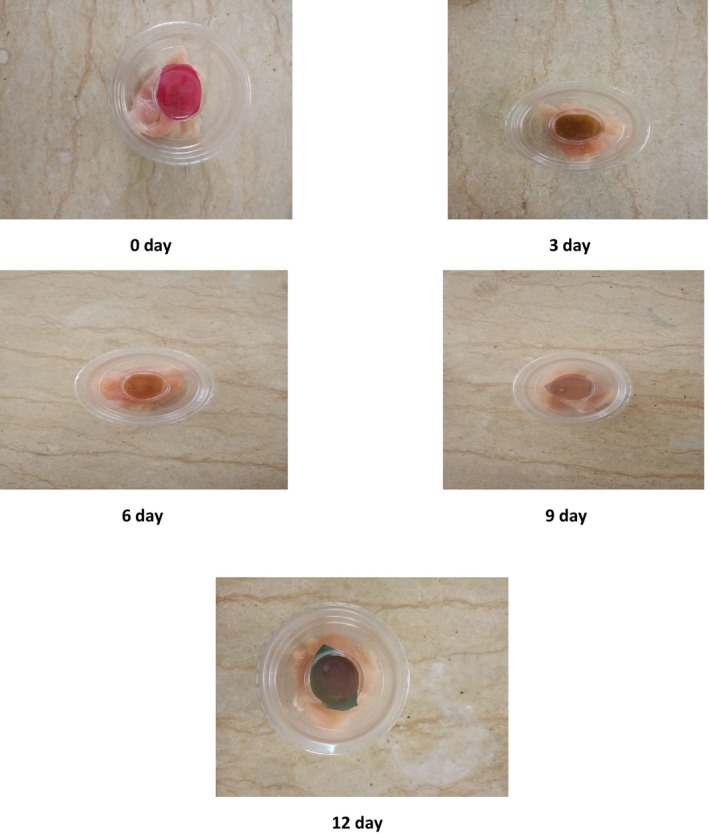
Visual changes in halochromic color of PVA film incorporated with *Kalanchoë blossfeldiana* anthocyanins during storage of chicken filet at 5°C.

Anthocyanins undergo pH‐dependent structural interconversion—flavylium cation (red, acidic) ⇄ quinoidal bases (purple) ⇄ chalcones (pale/yellow) ⇄ anionic forms (blue/green). Volatile bases and rising pH during spoilage shift the equilibrium, producing progressive hue changes. It was quantified as ΔE, aligning the visual readout with pH and TVB‐N (Figure 8).

As shown in Figure 8, the films exhibited distinct color variations over the storage period. The color of anthocyanins shifts in response to pH changes and ammonia emissions, which occur as a result of microbial activity and protein degradation during storage (Roy and Rhim 2021).

In the initial storage phase (0–3 days), when ammonia emission is minimal and the pH remains low, anthocyanins predominantly exist in their flavylium cation form, appearing red. This coloration indicates that the chicken filet remains fresh. As storage progresses and ammonia emissions begin to rise, leading to a shift toward neutral and alkaline conditions, the flavylium cation transitions into its quinonoidal base form, resulting in a brownish opaque color (Ahmad Alamer et al. 2025).

With further storage (6–9 days), the increasing alkalinity can degrade the anthocyanin structure, leading to a loss of pigmentation or the formation of chalcone derivatives, which appear colorless or pale yellow (Lema et al. 2022). By the later stages of storage (9–12 days), anthocyanins undergo further transformation into their anionic quinonoidal form, giving rise to blue or greenish‐blue hues (En et al. 2025).

These color changes serve as visual indicators of chicken filet freshness, with the transition from red to brown and ultimately to blue‐green aligning with progressive spoilage. The observed trends closely mirror those recorded for minced meat, confirming the potential of anthocyanin‐based films as freshness indicators for poultry products. It is important to note that the halochromic color change is irreversible under storage conditions, which limits the films to single‐use applications.

### 3.10 pH Of Chicken Filet During Storage

**FIGURE 9:**
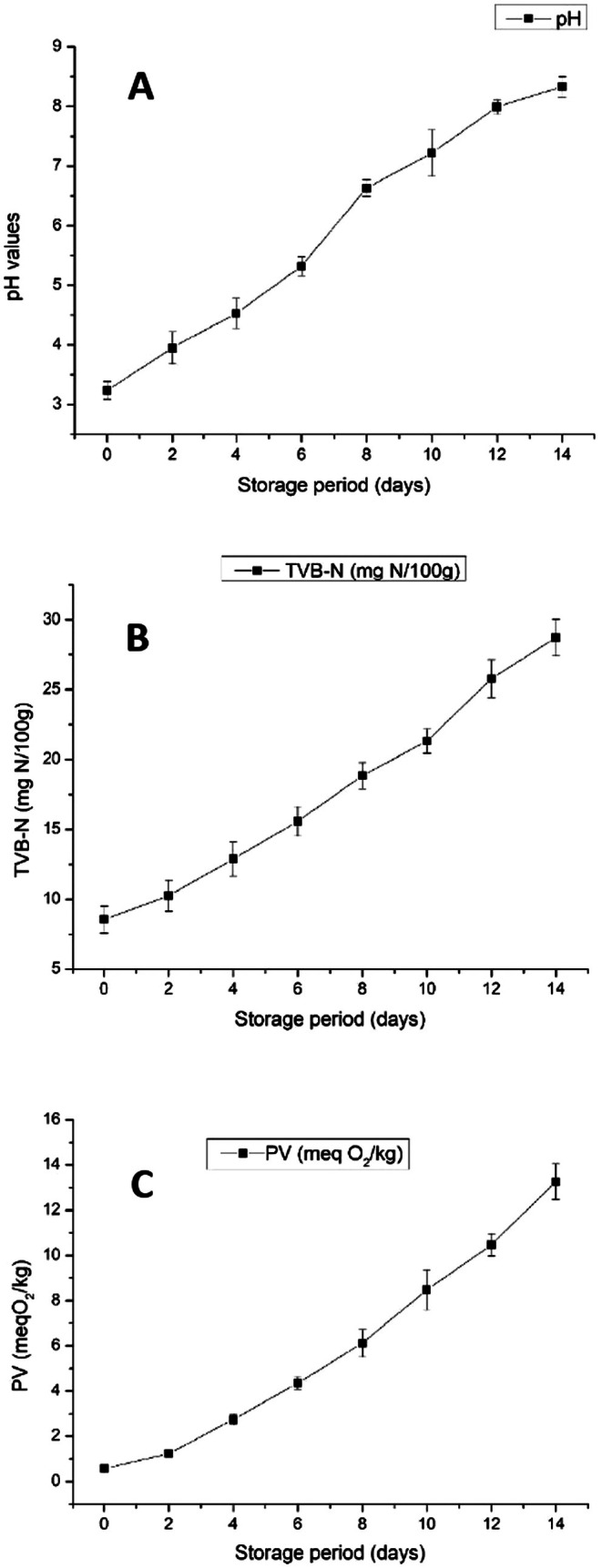
Changes in pH (A), total volatile basic nitrogen (TVB‐N) (B), and peroxide value (PV) (C) of chicken filet during storage at 5°C.

Figure 9A shows that the pH changes in chicken filet stored at 5°C over 14 days, showing a steady increase from approximately 3.2 to 8.5. This rising trend suggests progressive biochemical and microbial activity. The increase in pH is primarily attributed to the growth of spoilage bacteria, including Pseudomonas spp., Enterobacteriaceae, and *Brochothrix thermosphacta* , which produce basic nitrogenous compounds such as ammonia and volatile amines (Pellissery et al. 2020). Additionally, endogenous and microbial proteolysis leads to the breakdown of proteins into peptides and free amino acids, further elevating the pH (Abedi‐Firoozjah et al. 2022). Similar findings have been reported in other studies, such as those by (Katiyo et al. 2020), who observed that chicken stored at refrigeration temperatures exhibited a continuous pH increase due to microbial spoilage.

The increasing pH serves as a well‐established indicator of poultry spoilage. Generally, fresh chicken maintains a pH below 6, but as microbial activity progresses, the pH rises beyond 7, indicating the onset of spoilage (Wen et al. 2024). In comparison, (Rodríguez‐Calleja et al. 2012) found that chicken filets stored at 4°C exhibited a slower pH rise, reaching only 7.5 by day 14, likely due to better packaging conditions or initial bacterial load differences. The observed pH rise matched typical aerobic storage profiles.

Halochromic films provide a promising solution for real‐time freshness monitoring by visually indicating pH changes. These films, which change color based on pH variations, offer a non‐invasive method for assessing meat quality, reducing food waste, and ensuring consumer safety (Ismail and Huda 2024). Several studies have confirmed the effectiveness of halochromic films, such as those by (Ahmad Alamer et al. 2025), who demonstrated that halochromic‐based indicators incorporated with anthocyanins effectively detected spoilage in meat products. Compared to conventional freshness indicators like microbial plate counts or chemical assays, halochromic films provide a more accessible and real‐time method for quality assessment. The integration of such smart packaging technologies represents a significant advancement in food monitoring, offering a practical and cost‐effective solution for consumers and retailers.

### 3.11 TVB‐N Chicken Filet During Storage

Figure 9B illustrates the changes in total volatile basic nitrogen (TVB‐N) in chicken filet stored at 5°C over 14 days. TVB‐N is a widely used indicator of protein degradation and microbial spoilage in meat and fish, measuring the concentration of volatile nitrogenous compounds such as ammonia, trimethylamine, and dimethylamine. The results show a steady increase in TVB‐N from approximately 8 to over 30 mg N/100 g by day 14, indicating significant spoilage. This rise is attributed to proteolytic bacterial activity, particularly from Pseudomonas spp., Enterobacteriaceae, and *Shewanella putrefaciens* , which break down proteins into nitrogenous byproducts. Similar findings were reported by Baltić et al. (2017), who observed that TVB‐N levels in refrigerated chicken increased significantly over 14 days due to microbial metabolism.

Comparing with previous studies, the observed TVB‐N increase aligns with findings by Bekhit et al. (2021), who reported that TVB‐N levels rise steadily during refrigerated storage and are closely correlated with spoilage markers such as microbial growth and temperature across meat types. According to European food safety standards, TVB‐N levels above 25–30 mg N/100 g indicate meat spoilage and potential unsuitability for consumption (EC Regulation No 2074/2005). The present study's data suggest that the chicken filet exceeded the safe limit around day 12. The observed TVB‐N increase in aerobically stored chicken in this study parallels findings reported by Lei et al. (2023), who documented TVB‐N levels exceeding 30 mg N/100 g after 10–12 days at 4°C in aerobic conditions, while vacuum and MAP samples remained below spoilage thresholds. A recent review by Baltić et al. (2017) further supports these observations, highlighting that vacuum packaging and MAP can maintain TVB‐N values below critical spoilage limits for up to 20 days, reinforcing the significant role of packaging atmosphere in extending poultry shelf life. These trends reinforce the utility of a visual freshness indicator that correlates with standard spoilage markers (TVB‐N, pH).

### 3.12 Peroxide Value of Chicken Filet During Storage

Peroxide value is a primary indicator of lipid oxidation, representing the concentration of peroxides and hydroperoxides formed during the initial stages of oxidation. The results show a continuous increase in PV from 0.56 meq O_2_/kg at day 0 to 13.26 meq O_2_/kg at day 14, indicating progressive lipid oxidation over the storage period (Figure 9C). This increase is expected due to exposure to oxygen, the presence of unsaturated fatty acids, and microbial activity, all of which contribute to lipid degradation. Studies such as those by Narciso‐Gaytán et al. (2010); Pop et al. (2016) have reported similar trends in refrigerated poultry, where PV increased steadily during storage due to oxidative stress on lipids.

The observed increase in PV in this study is consistent with previous reports on the oxidative stability of chicken meat under different storage conditions. da Rocha et al. (2022) reported a gradual rise in peroxide value for wooden breast (WB) chicken meat, with PV increasing from 1.00 to 1.40 meq O_2_/kg after 7 days of refrigeration, and reaching up to 1.96 meq O_2_/kg during extended frozen storage at −18°C. Similarly, Alirezalu et al. (2022) found that chicken breast meat treated with calcium‐alginate coating and *Artemisia fragrans* essential oil exhibited significantly lower PV values (3.45 meq O_2_/kg) compared to uncoated controls (6.58 meq O_2_/kg) after 12 days of refrigerated storage. These findings highlight the influence of storage conditions and antioxidant‐rich coatings on lipid oxidation.

The continuous increase in PV indicates that oxidative spoilage is progressing and could impact the sensory quality of the chicken filet, leading to rancidity. This suggests that by day 12–14, the chicken filet in the present study might exhibit noticeable oxidative deterioration. Overall, the findings reinforce the importance of oxidation control strategies in maintaining poultry meat quality during storage.

## 4 Conclusion

Anthocyanin‐enriched PVA films exhibited enhanced hydrophilicity, optical responsiveness, and bioactivity, supporting their use as intelligent packaging materials. Incorporation of *Kalanchoë blossfeldiana* anthocyanins conferred strong antioxidant and antimicrobial effects and enabled clear, pH‐linked halochromic color changes that tracked poultry spoilage indicators (pH, TVB‐N). While higher pigment loadings reduced tensile and thermal stability, the films remained functionally effective for freshness monitoring. Because the chromatic transition is irreversible, the indicators are intended for single‐use applications—particularly in cold‐chain poultry packaging where moderate mechanical demands are acceptable and rapid visual readout is prioritized. In line with these findings, forthcoming studies will (i) map relative humidity (RH) stability (33%–97% RH, 25°C) with coupled mechanical (TS/EB), barrier (WVP), wettability (WCA), and color (ΔE*) readouts; (ii) quantify anthocyanin release kinetics from the PVA matrix into food simulants (PBS, 10% and 50% ethanol) by UV–Vis at A_520_, compute Mₜ/M∞, and fit Korsmeyer–Peppas models to relate transport to antibacterial efficacy; and (iii) complete targeted colorimetry—UV–Vis profiling across pH 1–12, film pH‐response in defined vapor/liquid buffers, and volatile NH_3_ headspace challenges—to establish sensitivity and detection thresholds. These efforts will refine end‐use guidelines and mechanistically link pH sensitivity and release behavior to antibacterial performance.

## Author Contributions

Mahmoud Younis and Diaeldin Omer Abdelkarim: Writing – review and editing, supervision, funding acquisition. Reham M. Kamel, Said El Harkaoui, Mahmoud Elsayed and Mohamed Abdin: Writing – original draft, project administration. Yasmin Salama, Mona Hussein Hassan, Mohamed Abdelbaset Salama and Mohamed Reda Badr: conceptualization, methodology, formal analysis, investigation, visualization, writing – original draft, writing – review and editing.

## Conflicts of Interest

The authors declare no conflicts of interest.

## Data Availability

The data that support the findings of this study are available from the corresponding author upon reasonable request.
